# Exploring a Novel Processing Algorithm to Improve Image Quality and Reduce X-Ray and Contrast Dose for Coronary Angiography: A Blinded Pilot Study

**DOI:** 10.1016/j.jscai.2026.105343

**Published:** 2026-05-12

**Authors:** Saba Assar, Mark Winkens, Kajo van der Marel, Roy van Pelt, Lindsay Johnson, Martijn S. van Mourik, Ragui Ibrahim, Philippe L’Allier, Lim Soo Teik, Leah Raj, William Nicholson, Michael Collins, Ajay J. Kirtane

**Affiliations:** a Columbia University Irving Medical Center/NewYork-Presbyterian Hospital, New York, New York; b Cardiovascular Research Foundation, New York, New York; c University of West Virginia, Morgantown, West Virginia; d ETZ Tweesteden, Tilburg, the Netherlands; e Philips Image Guided Therapy System, Best, the Netherlands; f Hôpital Pierre-Boucher, Longueuil, Quebec, Canada; g Montreal Heart Institute, Montreal, Quebec, Canada; h National Heart Centre Singapore, Singapore; i Emory Healthcare, Atlanta, Georgia

**Keywords:** contrast exposure, coronary angiography, radiation exposure, x-ray acquisition algorithm

## Abstract

**Background:**

With advancements in percutaneous coronary intervention, clinicians face the challenge of balancing the need for high-quality imaging with the imperative to minimize radiation exposure and iodinated contrast use, particularly in complex procedures.

**Methods:**

We conducted a pilot study of 50 patients undergoing diagnostic coronary angiography in which each patient received 2 sequential angiograms, one using conventional image acquisition and processing protocols and the other acquired and processed using a novel algorithm, with a reduction in radiation, iodine content, or both. Angiographic runs were analyzed offline by 5 interventional cardiologists who independently reviewed the images, blinded to the image acquisition protocol. The study assessed image quality between the 2 acquisition protocols as well as interobserver variability to measure consistency in scoring among the evaluators.

**Results:**

The mean age of the patients was 63 ± 10 years (74% male). The mean body mass index was 27.8 ± 5.0 kg/m^2^, and 8 of 50 (16%) had a glomerular filtration rate <60 mL/min. When comparing 5-point image quality scores, the novel algorithm scored higher than the predicate comparator (on average, 1 point higher for the novel algorithm images over ClarityIQ images [*P* < .01]). In a blinded side-by-side comparison, the novel algorithm was preferred in 214 of 220 ratings and was found to be superior to the predicate comparator for both standard radiation and lower radiation cases. The interobserver correlation of preference between the protocols among the 5 readers ranged from 0.53 to 0.67, with an average of 0.61.

**Conclusions:**

These data suggest that the use of novel x-ray acquisition algorithms can improve image quality while maintaining or lowering radiation or iodine dose. The prospective evaluation of these algorithms within routine clinical practice is warranted in future clinical studies.

Over the past 4 decades, there have been significant advancements in the field of percutaneous coronary intervention (PCI), facilitating treatment of increasingly complex disease in higher risk patients.^1^ Increasing procedural and patient complexity can lead to longer procedure times, which can lead to increased contrast usage and radiation dose. However, especially in high-complexity procedures, clinicians must balance limiting radiation dose and contrast volume with the need to preserve image quality to safely perform PCI procedures.

One concern in longer, more complex procedures is the higher radiation dose due to prolonged x-ray usage.^2^, ^3^, ^4^, ^5^, ^6^ Higher radiation doses affect both the patient and all staff working inside the procedure room.^7^ There has been a growing awareness of the adverse effects of radiation, and although shielding and equipment improvements contribute to reducing exposure,^8^ there is still a need to reduce dose, especially in longer, more complex procedures. The main challenge in reducing radiation dose is that image quality is fundamentally related to the radiation dose used in image acquisition.^9^ Clinicians can lower radiation dose on existing x-ray imaging systems, but this tends to result in lower image quality, which may limit the clinician’s ability to perform the procedure safely or efficiently. Another complication of PCI is contrast-induced acute kidney injury (CI-AKI), which is associated with increased morbidity, mortality, and health care costs. CI-AKI is associated with the amount of administered iodine contrast.^8^

In light of these challenges, novel optimized x-ray processing algorithms have been developed to reduce both x-ray radiation dose as well as the dose of iodinated contrast while maintaining image quality.^10^^,^^11^ One such system, ClarityIQ (Philips Healthcare), uses dose-reduction algorithms combined with an optimized acquisition chain to achieve x-ray dose reduction while preserving image quality^11^ and is widely used in current clinical practice. A subsequent iteration of ClarityIQ, referred to herein as the novel algorithm (SmartIQ, Philips Healthcare) has been further optimized with the goal of improving image quality while reducing both radiation dose and iodinated contrast dose. In this proof-of-concept study, we assessed diagnostic image quality of coronary angiogram acquisitions processed with the novel algorithm compared with ClarityIQ.

## Methods

The primary aim of the study was to compare the diagnostic image quality of coronary angiogram acquisitions processed with the novel algorithm compared with standard ClarityIQ using specific acquisition settings. The study included 50 patients who underwent diagnostic coronary angiography or PCI between May and September 2021 at the Elisabeth TweeSteden Hospital in Tilburg, the Netherlands. The Dutch medical ethical commission (MECT) approved this study and patient consent was obtained before enrollment. All studies were performed on a Philips Azurion 7 C12 x-ray system equipped with both ClarityIQ as well as the novel algorithm. The novel algorithm processes spatial and temporal image signal characteristics to substantially enhance visibility of iodine contrast-filled coronaries. This facilitates improved imaging with reductions in radiation or iodine contrast dose under difficult conditions (eg, steep C-arm angulations).

For each patient, one coronary angiogram was performed using a selected ClarityIQ setting, and this was immediately followed by a second coronary angiogram per case, which was processed by the novel algorithm. At the start of each procedure, iodine contrast dose and system radiation settings were selected by the operator based on the patient’s baseline characteristics, which accounted for body mass index and kidney function. For ClarityIQ, radiation dose setting options were medium or low, and the novel algorithm options were medium, low, and ½ low as follows: the radiation dose settings for ClarityIQ and the novel algorithm are the same across the 2 systems for a given designation (eg, medium and low). For example, the ClarityIQ’s medium dose settings are equivalent to the novel algorithm’s medium. The ½ low setting is only available for the novel algorithm and is 50% of the radiation of the low setting of ClarityIQ.

**Figure 1:**
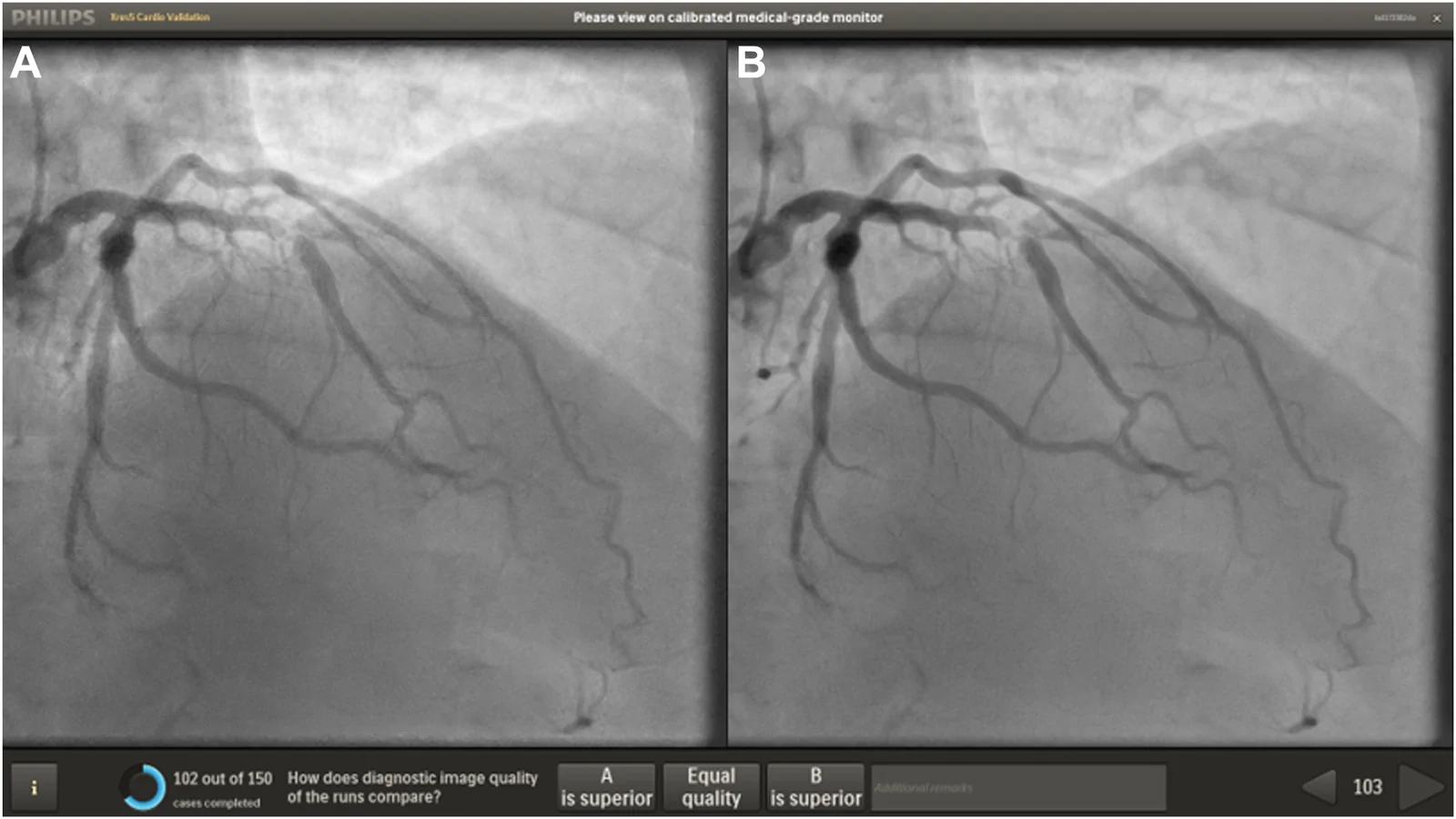
**Example of reader comparison task.** In this case, (**A**) was processed with ClarityIQ and was acquired with medium radiation and standard iodine dosage, while (**B**) was processed with the novel algorithm and was acquired with the medium radiation dose and standard iodine dosage.

**Figure 2:**
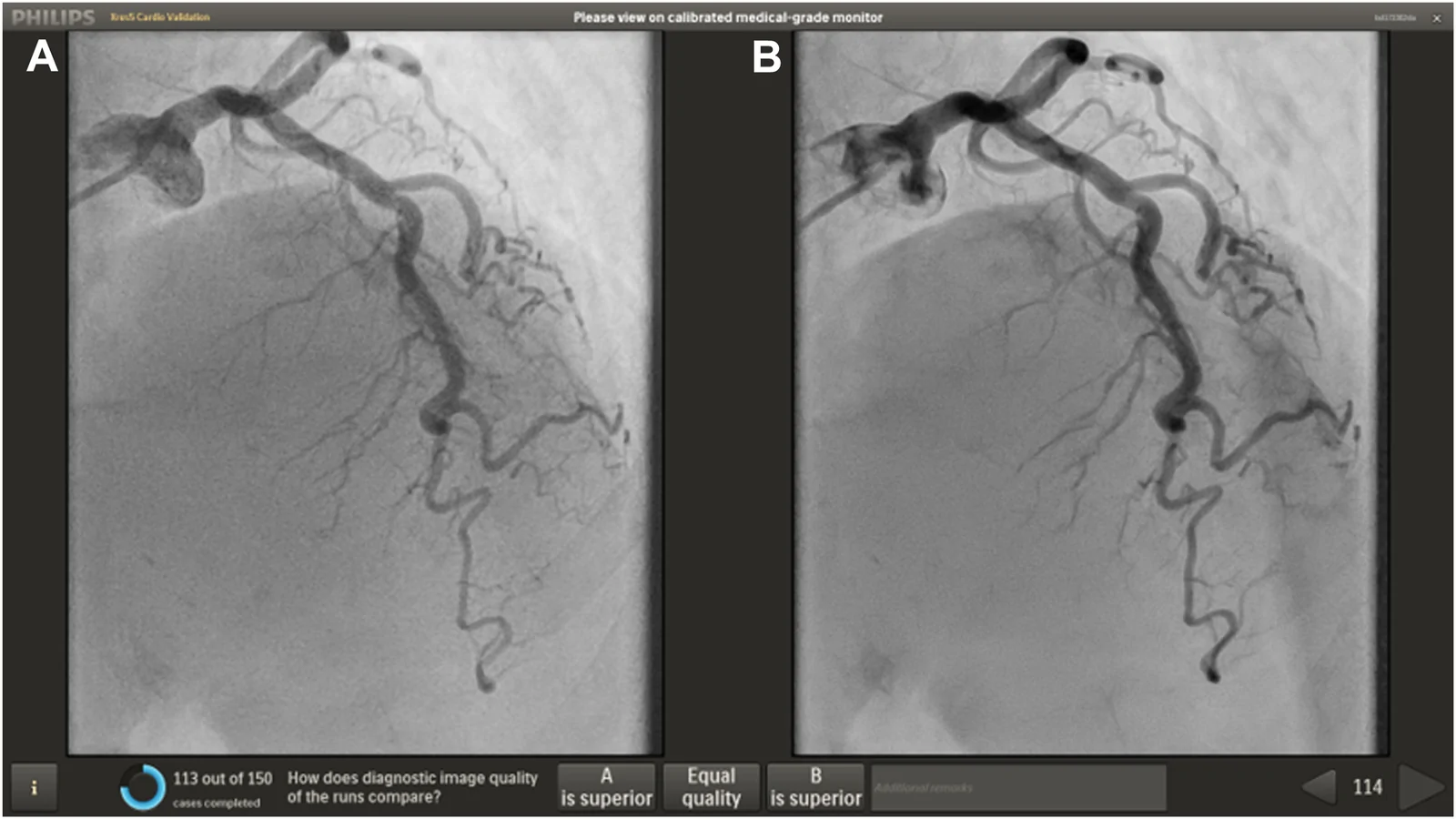
**Example of reader comparison task.** In this case, (**A**) was processed with ClarityIQ and was acquired with low radiation and standard iodine dosage, while (**B**) was processed with the novel algorithm and was acquired with the ½ low radiation dose and standard iodine dosage.

The operator chose to match the radiation dose (Table 1, “Standard”) with either medium or low acquisitions for both algorithms or to have a reduced radiation comparison (Table 1, “Reduced Radiation”) between low (ClarityIQ) and ½ low (novel algorithm). Figure 1 demonstrates an example of side-by-side comparison of medium radiation setting of both ClarityIQ and the novel algorithm using standard iodine dosage in administered contrast. Similarly, Figure 2 shows an example of a standard iodine angiogram comparison where ClarityIQ was acquired with low radiation dose, and the novel algorithm was acquired with ½ low.

**Table 1:** Selected ClarityIQ acquisition settings and corresponding the novel algorithm coronary angiogram settings in individual patients for Iodine doses of 320 mg/mL

	ClarityIQ radiation dose	Novel algorithm radiation dose	No. of cases
Standard	Medium	Medium	4
	Low	Low	25
Reduced radiation	Low	½ Low	15

**Figure 3:**
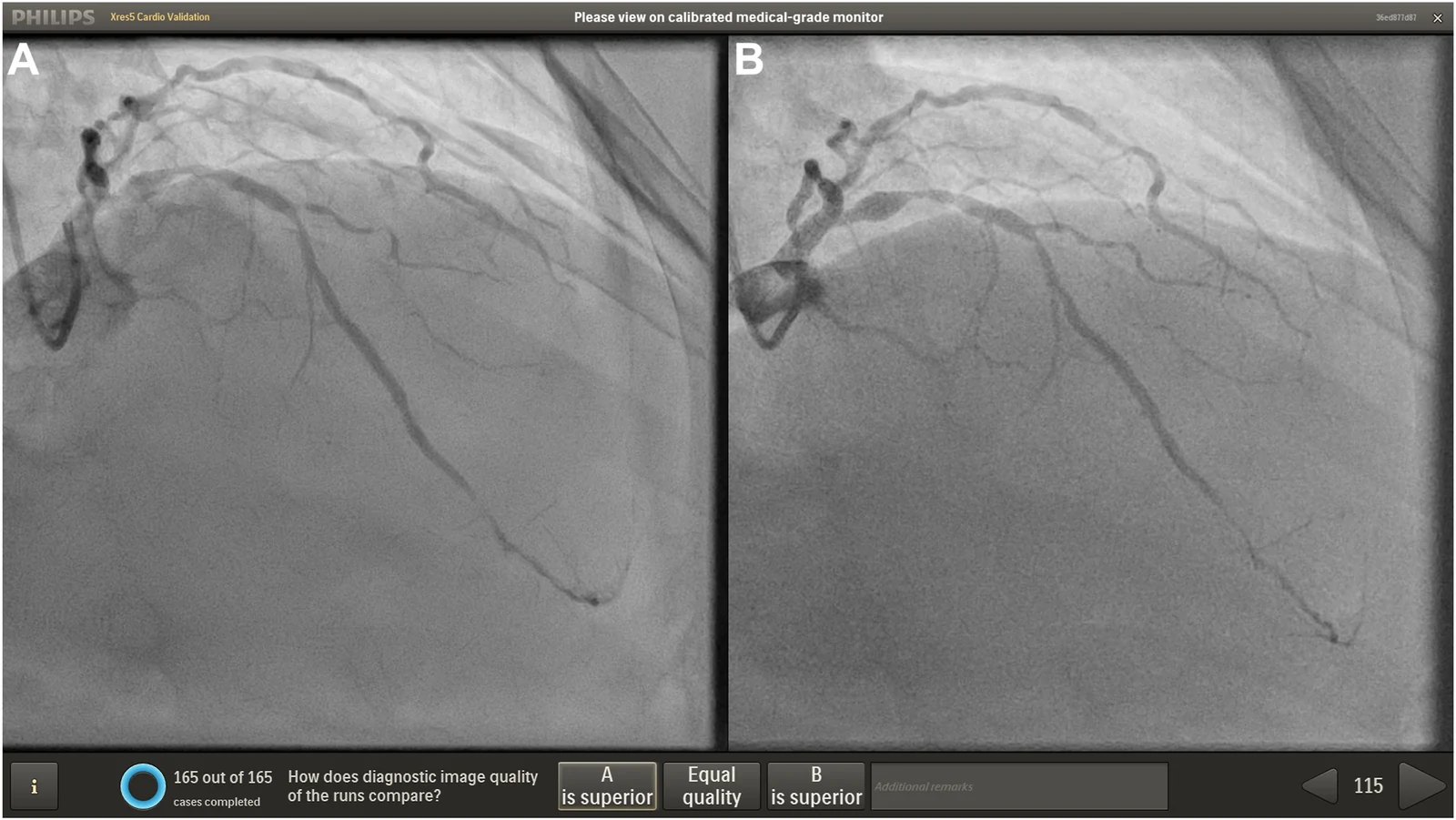
**Example of reader comparison test.** In this case, (**A**) was processed with the novel algorithm and was acquired with low radiation and lower dosage of iodine in contrast (235 mg/mL), while (**B**) was processed with ClarityIQ and was acquired at low radiation and standard dosage of iodine in contrast (320 mg/mL).

For iodine contrast dose, the standard dose was defined as 320 mg/mL iodixanol (Visipaque), and any lower concentration of iodine was considered a reduced iodine dosage. These options resulted in a variety of acquisition combinations as the same settings were not required to be used for the 2 sequential angiograms. The primary analysis dataset includes the 44 cases with standard iodine dose of 320 mg/mL (Table 1). Only a limited number of cases (6 cases) used reduced iodine dosage with the novel algorithm acquisition; these are described in Supplemental Tables S1 and S2; injected iodine contrast dose in these ranged from 190 to 270 mg/mL. Figure 3 demonstrates a side-by-side comparison, both acquired at low dose settings, with ClarityIQ obtained with standard contrast (320 mg/mL) and the novel algorithm using reduced iodine content of contrast (235 mg/mL).

Paired angiography runs were assessed in an offline reader study in which 5 interventional cardiologists from different institutions independently and blindly reviewed the images. Readers were unaware of image acquisition settings (radiation dose, iodine dose, and processing algorithm). Participating centers were Hôpital Pierre-Boucher (Longueuil, Quebec, Canada), Montreal Heart Institute (Montreal, Quebec, Canada), Emory Healthcare (Atlanta, Georgia), Columbia University Irving Medical Center/NewYork-Presbyterian Hospital (New York, New York) and National Heart Center Singapore (Singapore). First, readers assessed diagnostic image quality, where all images were evaluated individually, in random order, mixing both the ClarityIQ and the novel algorithm images. The images were rated on a scale with grades 1 to 5 assigned to each image, as defined in Table 2. Next, to determine preference for ClarityIQ or the novel algorithm, the same-patient paired angiograms (one ClarityIQ, one with the novel algorithm) were presented to evaluators in a side-by-side, blinded, and randomized order of display. Readers rated whether a specific image was superior or if they were equal in quality.

**Table 2:** Scores and definitions for image quality assessment

Image quality grade	Grade description
1	Very poor – unsatisfactory for diagnosis.
2	Mediocre – artery outline not sharp. Potential for missing features.
3	Average – fair vessel visualization, sharp coronary arteries (smooth vessel wall), sufficient for diagnosis but requires longer time to interpret.
4	Good – all branches visible with good discrimination of crossing arteries.
5	Excellent – superior visualization of the vasculature.

### Statistical analysis

For the 44 single angiograms with standard iodine cases in Table 1, statistical analysis was performed to determine differences in image quality scoring and superiority in preference rating. To determine if diagnostic image quality scores varied between ClarityIQ and the novel algorithm, the Mann-Whitney U test was used. Reader diagnostic scores were collapsed to a median score per image for ClarityIQ and the novel algorithm. These paired median scores were then used as inputs to the Mann-Whitney *U* test. Next, an exact binomial test was used to assess the superiority of the novel algorithm over ClarityIQ, comparing the number of preferences for the novel algorithm against the number of preferences for ClarityIQ or ties, based on preference scores from all observers. The data were additionally stratified by overall radiation dose (29 patients with standard radiation dose and 15 patients with reduced radiation dose).

Finally, interobserver variation was assessed for the 5 physicians. The level of agreement between panel members was evaluated using Spearman correlation using the image quality rating scores for each reader and both ClarityIQ and the novel algorithm.

## Results

Baseline demographics for the subjects are presented in Table 3, with participants having an average age of 62 ± 10 years and a body mass index of 27.4 ± 4.6 kg/m^2^. Of 44 participants, 35 presented with stable coronary artery disease, 1 presented with non-ST elevation myocardial infarction, and 8 were undergoing screening for other procedures. In terms of the interventions performed, 17 subjects received PCIs, whereas 27 subjects had diagnostic-only procedures. Standard acquisition with 320 mg/mL iodine and either medium or low radiation dose setting was selected in 29 patients (Table 1). In 15 patients, ClarityIQ had low radiation dose and with the novel algorithm had ½ low dose.

**Table 3:** Baseline demographics

Demographic information	N = 44
Age, y	62 ± 10
Male sex	33 (75)
BMI, kg/m^2^	27.4 ± 4.6
GFR <60 mL/min	5 (11)

Values are mean ± SD or n (%).

BMI, body mass index; GFR, glomerular filtration rate.

**Figure 4:**
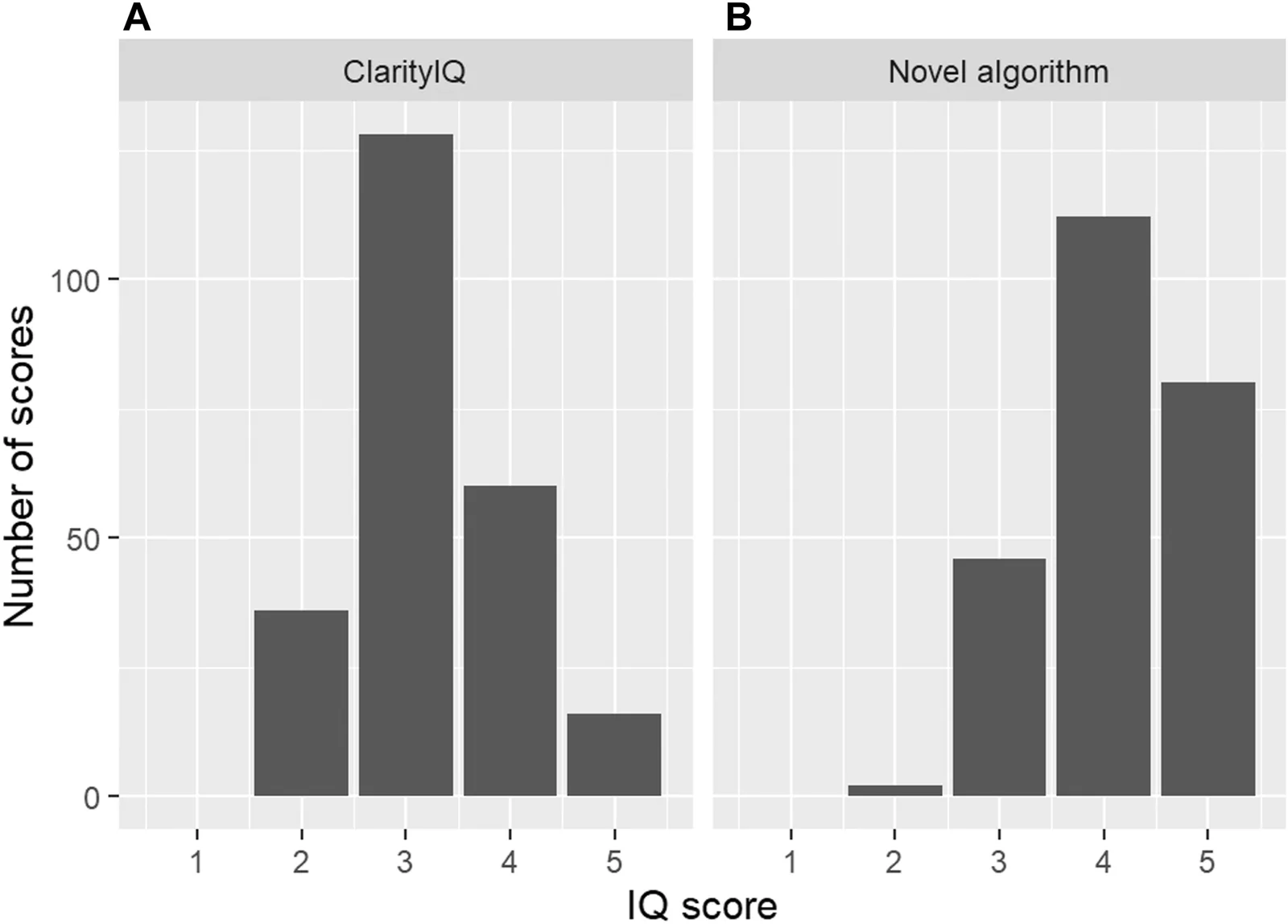
**Bar graphs showing a histogram of image quality scores for (A) ClarityIQ and (B) the novel algorithm for all ratings in**Table 1.

**Figure 5:**
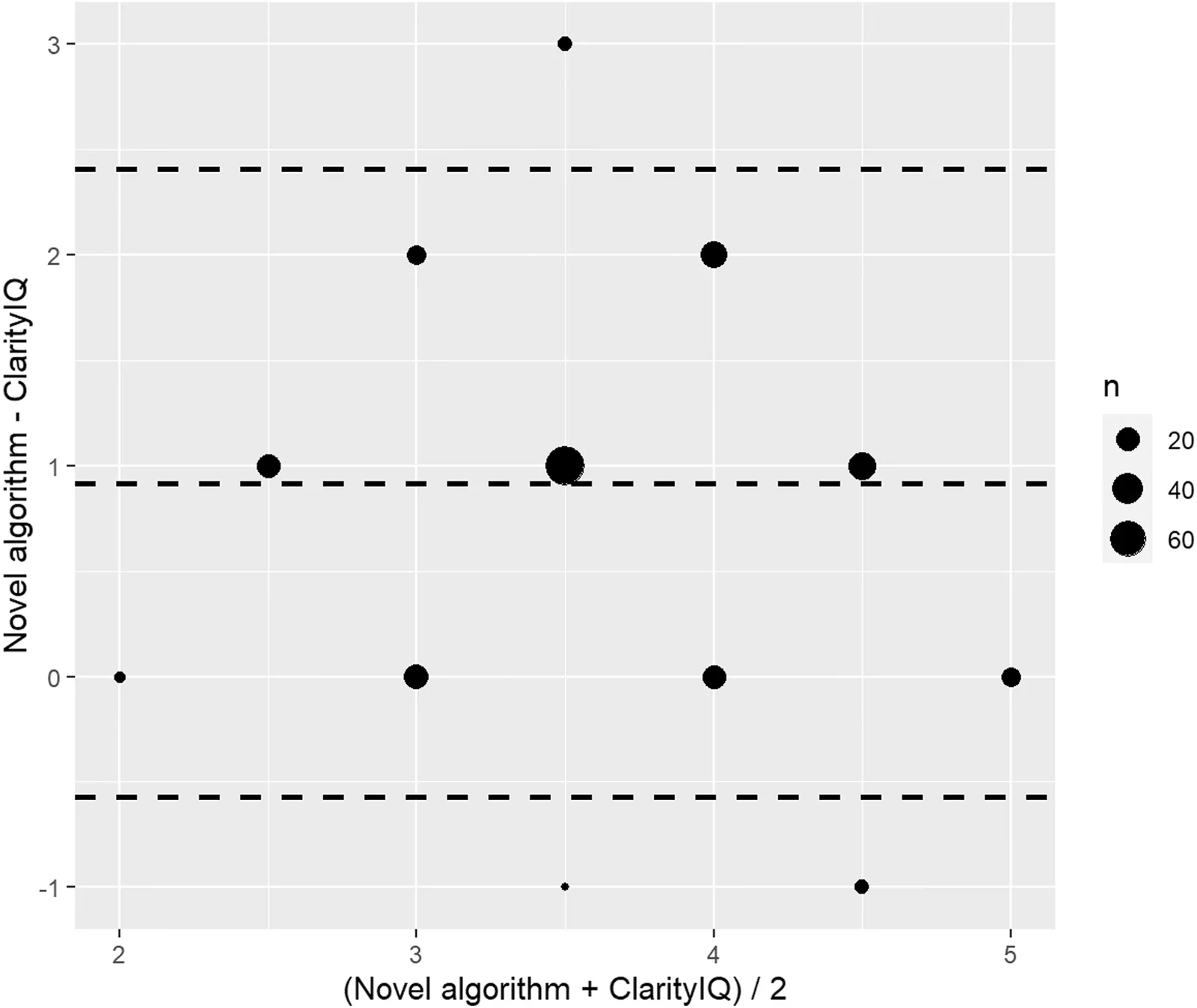
**Bland-Altman plot of the difference of image quality ratings which shows the improved image quality of the novel algorithm over ClarityIQ**.

**Figure 6:**
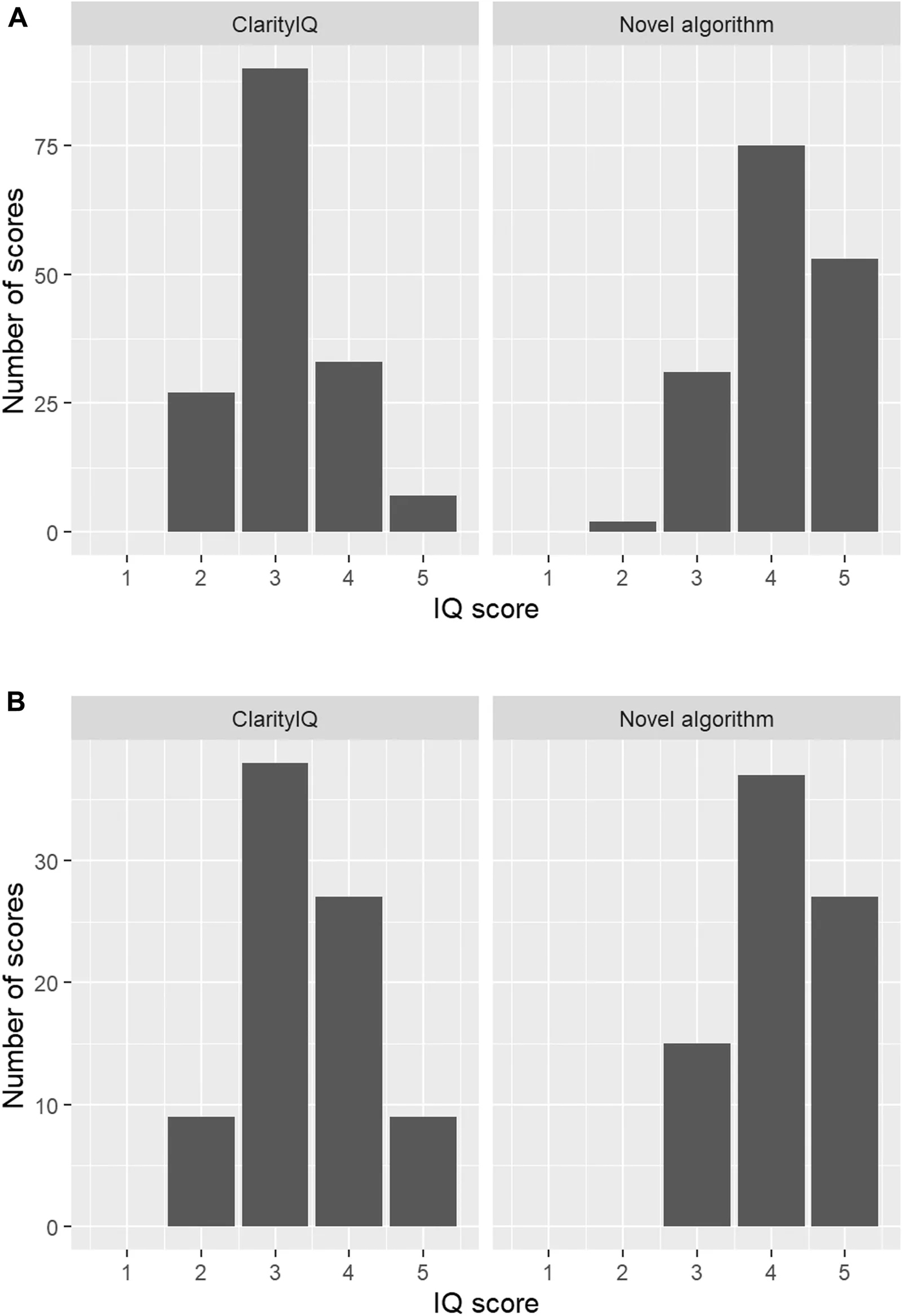
**Bar graphs showing a histogram of image quality scores for ClarityIQ and the novel algorithm divided into standard radiation (A) and reduced radiation (B) ratings**.

Overall, the novel algorithm received higher reader scores compared to ClarityIQ (Figure 4). Scores were higher by 1 point for the novel algorithm images over ClarityIQ images (Mann-Whitney *U* test, *P* < .01; 95% CI, 1.00-1.50). The differences between scores are visualized in a Bland-Altman plot (Figure 5). Scores were further stratified by standard radiation and reduced radiation doses, as demonstrated in Figure 6.

**Figure 7:**
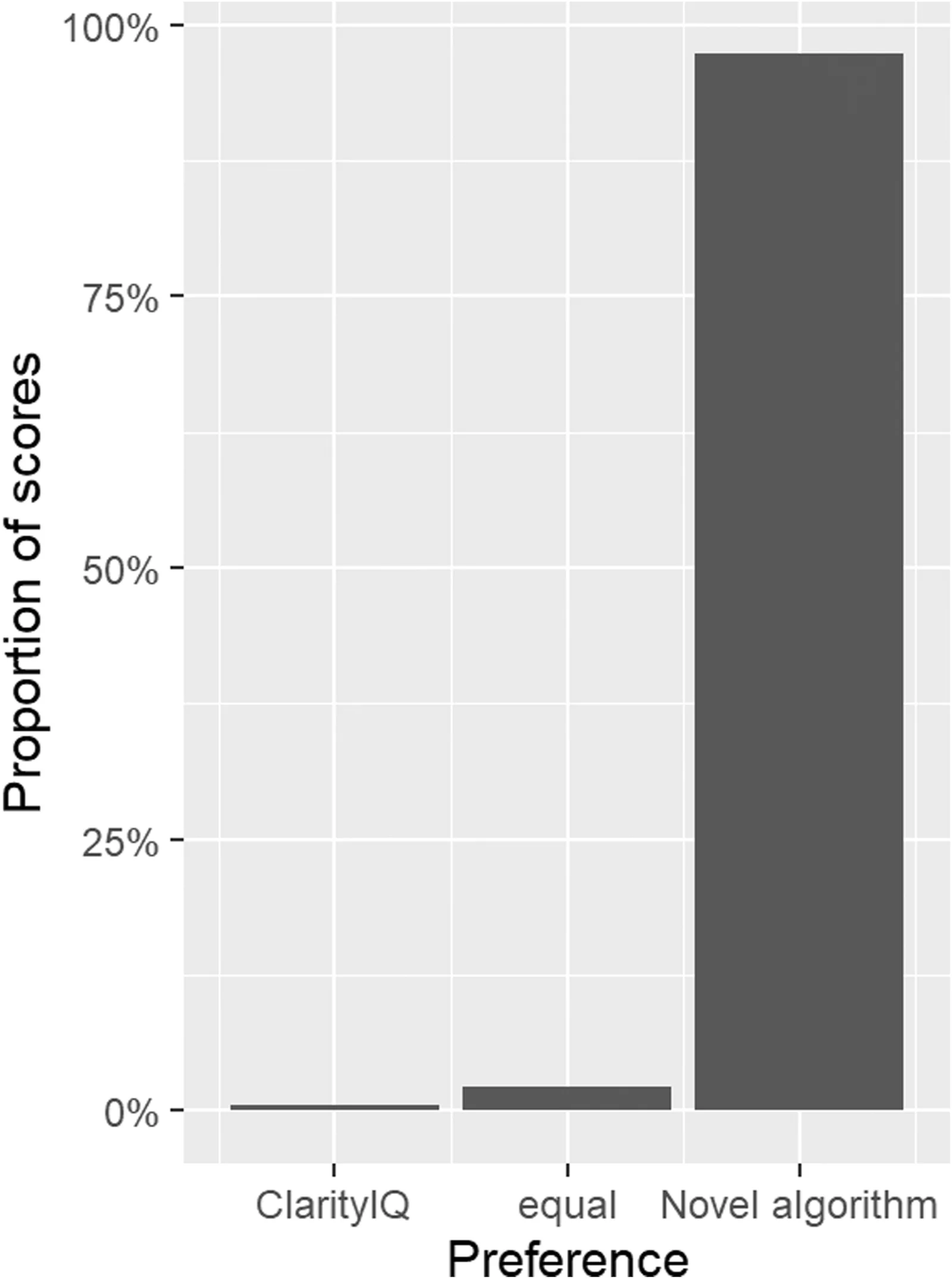
**Bar graphs showing proportion of preference during direct comparison for all ratings. ClarityIQ was chosen in only 0.4% of cases**.

The blinded side-by-side comparison showed the novel algorithm was preferred in 214 of 220 ratings (Figure 7). The novel algorithm was found to be superior to ClarityIQ (exact binomial, *P* < .01; 95% CI, 0.94-0.99). Only in one instance did a single reader (of 5) rate ClarityIQ as the preferred image, which was a case in which ClarityIQ was a low radiation acquisition while the novel algorithm was ½ low dose. In stratified analysis, the novel algorithm was determined to be superior to ClarityIQ for both standard radiation with the novel algorithm being preferred 143 of 145 times (98.6%, 95% CI: 0.95-1.00), as well as lower radiation cases, with the novel algorithm being preferred in 71 of 75 ratings (94.7%; 95% CI, 0.87-0.98). For the same 44 cases, the interobserver correlation between the 5 readers ranged from 0.53 to 0.67, with an average of 0.61 (Table 4), or a moderate to strong correlation.^12^

**Table 4:** Interobserver correlation results using Spearman correlation between observers

Reader	1	2	3	4	5
1		0.67	0.61	0.55	0.67
2			0.57	0.53	0.60
3				0.63	0.65
4					0.59
5					

Six patients had protocols with the novel algorithm having reduced iodine compared with those of the corresponding ClarityIQ angiogram, as shown in Supplemental Tables S1 and S2. Three of the 6 patients had glomerular filtration rate <60 mL/min. In 2 of these cases, all 5 readers unanimously preferred the novel algorithm over ClarityIQ. In 3 of the cases, 3 or more readers rated the novel algorithm better than ClarityIQ, and in the final case, 3 readers rated the novel algorithm equal to that of ClarityIQ.

## Discussion

In this pilot study, we aimed to assess the diagnostic image quality of coronary angiogram acquisitions processed with a novel algorithm for image acquisition and visualization compared with standard of care (using ClarityIQ). Image quality scores and preference ratings indicated that the novel algorithm produced increased image quality relative to ClarityIQ, both when radiation dose was matched (standard radiation) and at a lower dose than ClarityIQ (reduced radiation) (Central Illustration). Although this study is limited with regard to the number of subjects as well as comparison of only 1 angiogram per participant, the novel algorithm has the potential to give clinicians more flexibility in image acquisitions by allowing for improved image quality with either the same amount or a lesser dose of radiation.

**Central Illustration:**
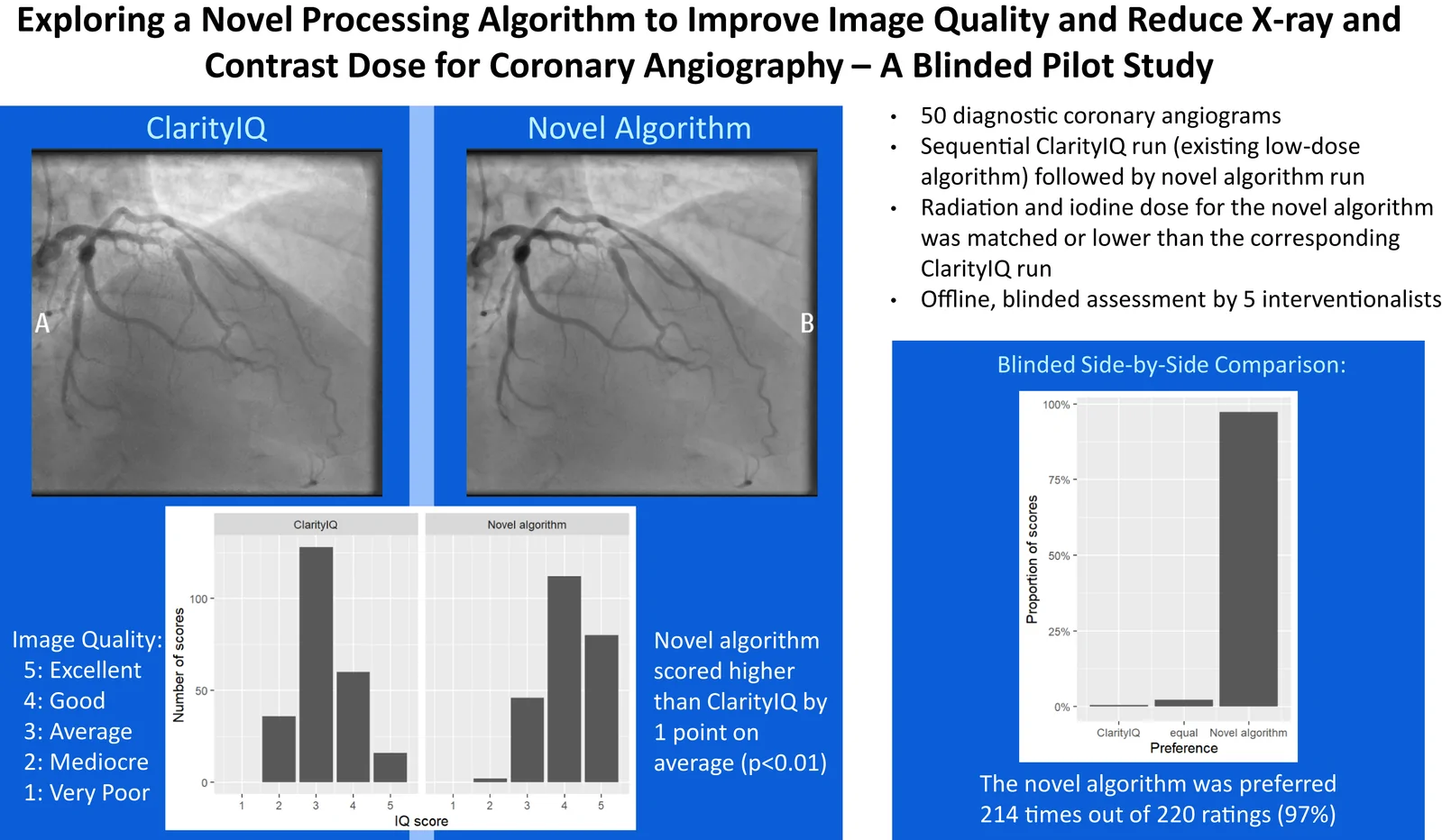
**Exploring a novel processing algorithm to improve image quality and reduce x-ray and contrast dose for coronary angiography – a blinded pilot study.**

If these findings can be further validated on a larger scale, they have important implications that extend beyond improved image quality and include enhanced procedural safety as well as efficiency. The ability to improve image quality at lower radiation dose can reduce total radiation exposure for both the patients as well as the health care team. This is particularly vital for members of the interventional team who accumulate radiation exposure over the span of their careers. Furthermore, the consistently higher image quality with the new algorithm may decrease the need for repeat acquisitions for suboptimal visualization, potentially improving procedural efficiency. This may be especially useful in cases with steep angulations or challenging anatomy.

Beyond offering the potential for improved or maintained image quality at lower radiation doses, the potential of the novel algorithm in facilitating low-iodine acquisitions may be a strategy to address the risk of CI-AKI in certain patient populations. In conventional practice, using lower iodine levels can result in poor image quality and compromise definitive diagnosis or treatment. The use of iodine contrast-sparing techniques including ultralow-contrast PCI^13^ as well as procedural tools integrated within the x-ray system, such as dynamic coronary road mapping,^14^ have been shown to reduce the amount of administered iodine contrast during PCI procedures, thereby potentially lowering the risk of CI-AKI. The novel algorithm studied in this report also aims to improve the image quality to decrease the necessary iodine contrast utilized. Although the novel algorithm appeared to be preferred to ClarityIQ in the 6 cases in which a lower iodine dose was used, it is difficult to draw firm conclusions from this small sample size. If these results are replicated in a larger series of patients, this would potentially allow clinicians even more flexibility in lowering the amount of iodine contrast administration while still maintaining desirable image quality.

### Limitations

Although the results of the study are promising, there are several important limitations and considerations. First, the results are based on a single coronary angiogram comparison and cannot be directly extrapolated to full procedures. Second, a large portion of radiation from coronary procedures arises from fluoroscopy, and utilizing this algorithm for cineangiograms only will therefore have an unknown effect on the total procedure radiation dose. Third, the novel algorithm is compared to its predecessor from the same vendor, and therefore no conclusion can be made in comparison to other x-ray processing algorithms. Finally, the analyses employed semiquantitative assessments of diagnostic image quality. This is also reflected in the moderate interobserver correlation in image quality scores; however, on average, readers still agreed that 97% of the runs processed with the novel algorithm were superior to ClarityIQ. Future larger-scale studies, including multicenter trials, would help to confirm these trends.

## Conclusions

The results of this study suggest that a novel algorithm in image acquisition and processing can further improve diagnostic image quality compared to its predecessor, ClarityIQ, even in challenging cases where lower doses of radiation, iodine, or a combination of both were used.

## CRediT authorship contribution statement

**Saba Assar:** Writing – original draft. **Mark Winkens:** Conceptualization, Funding acquisition. **Kajo van der Marel:** Formal analysis, Investigation, Project administration. **Roy van Pelt:** Formal analysis, Investigation, Project administration. **Lindsay Johnson:** Investigation, Methodology, Project administration. **Martijn S. van Mourik:** Formal analysis, Investigation, Project administration. **Ragui Ibrahim:** Data curation, Investigation. **Philippe L’Allier:** Data curation. **Lim Soo Teik:** Data curation, Investigation. **Leah Raj:** Data curation, Investigation. **William Nicholson:** Conceptualization, Supervision. **Michael Collins:** Conceptualization, Supervision. **Ajay J. Kirtane:** Conceptualization, Supervision, Writing – original draft.

## Declaration of competing interest

Kajo van Marel, Roy van Pelt, Lindsay Johnson, and Martijn van Mourik are employees of Philips. Philippe L'Allier reported minor consulting from Philips. William Nicholson reported research grants from Philips. Ajay J. Kirtane reported institutional funding to Columbia University and/or Cardiovascular Research Foundation from Abbott Vascular, Amgen, Bolt Medical, Boston Scientific, CathWorks, Concept Medical, Cordis, Idorsia, Janssen, Magenta Medical, Medtronic, Neurotronic, Philips, ReCor Medical, Supira, and Teleflex. In addition to research grants, institutional funding includes fees paid to Columbia University and/or Cardiovascular Research Foundation for consulting and/or speaking engagements in which Dr Kirtane controlled the content. Ajay J. Kirtane also reported equity options in Airiver, Aeroflow, Bolt Medical, Lumen Medical, Roon, Tonic Medical; consulting fees from Sonivie; travel expenses/meals from Abbott Vascular, Acist, Abiomed, Amgen, Angiodynamics, Biotronik, Boston Scientific, CathWorks, Chiesi, Concept Medical, Edwards Lifesciences, Kiniksa Pharmaceuticals, Medtronic, Novartis, Janssen, Philips, ReCor Medical, Shockwave, and Supira. Saba Assar, Mark Winkens, Ragui Ibrahim, Lim Teik, Leah Raj, and Michael Collins have no competing interests to declare.

## Footnotes

To access the supplementary material accompanying this article, visit the online version of the *Journal of the Society for Cardiovascular Angiography & Interventions* at 10.1016/j.jscai.2026.105343.
