# Supplementary material for: Exploring a Novel Processing Algorithm to Improve Image Quality and Reduce X-Ray and Contrast Dose for Coronary Angiography: A Blinded Pilot Study
Source: J Soc Cardiovasc Angiogr Interv. 2026 May 12;5(6):105343. doi: 10.1016/j.jscai.2026.105343 (PMC13550899; doi:10.1016/j.jscai.2026.105343)
Supplement: Supplementary file 1 [file mmc1.docx]

**Supplemental Material**

**Supplemental Table S1.** Demographics of low-iodine acquisition subjects.

| Demographic Information | Low Iodine  n=6 |
| --- | --- |
| Age (y, mean, SD) | 67 ± 10 |
| Male sex (n, %) | 4 (67%) |
| BMI (kg/m^2^) | 31.2 ± 6.7 |
| GFR<60 mL/min | 3 (50%) |

**Supplemental Table S2.** The selected ClarityIQ acquisition settings and the corresponding the novel algorithm coronary angiogram settings for six patients who underwent reduced Iodine administration for the novel algorithm coronary angiogram.

|  | **Radiation Dose** | | **Iodine Dose (mg/mL)** | | **Number of Cases** |
| --- | --- | --- | --- | --- | --- |
| **Reduced Iodine** | **ClarityIQ** | **the novel algorithm** | **ClarityIQ** | **the novel algorithm** |  |
|  | Low | Medium | 320 | 190 | 1 |
|  | Medium | Medium | 320 | 235 | 1 |
|  | Medium | Medium | 320 | 270 | 1 |
|  | Medium | Medium | 320 | 190 | 2 |
|  | Low | Low | 320 | 235 | 1 |
